# The effect of relative humidity on CaCl_2_ nanoparticles studied by soft X-ray absorption spectroscopy

**DOI:** 10.1039/d0ra08943e

**Published:** 2021-01-07

**Authors:** Abdul Rahman Abid, Maximilian Mailhiot, Nacer Boudjemia, Eetu Pelimanni, Aleksandar R. Milosavljević, Clara-Magdalena Saak, Marko Huttula, Olle Björneholm, Minna Patanen

**Affiliations:** a Nano and Molecular Systems Research Unit, Faculty of Science, University of Oulu P.O. Box 3000 90570 Oulu Finland abdul.abid@oulu.fi +358 46 9691089; b Molecular and Condensed Matter Physics, Uppsala University Ångströmlaboratoriet 752 37 Uppsala Sweden; c SOLEIL Synchrotron Facility L'Orme des Merisiers, BP 48 91190 Saint-Aubin France

## Abstract

Ca- and Cl-containing nanoparticles are common in atmosphere, originating for example from desert dust and sea water. The properties and effects on atmospheric processes of these aerosol particles depend on the relative humidity (RH) as they are often both hygroscopic and deliquescent. We present here a study of surface structure of free-flying CaCl_2_ nanoparticles (CaCl_2_-NPs) in the 100 nm size regime prepared at different humidity levels (RH: 11–85%). We also created mixed nanoparticles by aerosolizing a solution of CaCl_2_ and phenylalanine (Phe), which is a hydrophobic amino acid present in atmosphere. Information of hydration state of CaCl_2_-NPs and production of mixed CaCl_2_ + Phe nanoparticles was obtained using soft X-ray absorption spectroscopy (XAS) at Ca 2p, Cl 2p, C 1s, and O 1s edges. We also report Ca 2p and Cl 2p X-ray absorption spectra of an aqueous CaCl_2_ solution. The O 1s X-ray absorption spectra measured from hydrated CaCl_2_-NPs resemble liquid-like water spectrum, which is heavily influenced by the presence of ions. Core level spectra of Ca^2+^ and Cl^−^ ions do not show a clear dependence of % RH, indicating that the first coordination shell remains similar in all measured hydrated CaCl_2_-NPs, but they differ from aqueous solution and solid CaCl_2_.

## Abstract 2

Hydration state and surface composition of free-flying nanoparticles originating from aerosolized solutions of CaCl_2_ and CaCl_2_ and Phenylalanine are probed using X-ray absorption spectroscopy.

## Graphical abstract

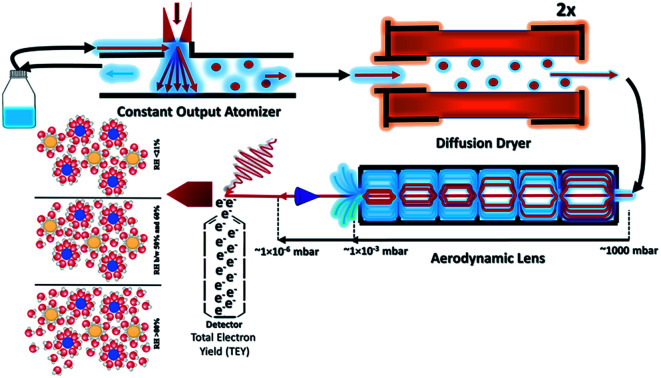

## 1 Introduction

Inorganic ions and salts are essential for life on Earth. They play key roles in biological processes of living organisms, *e.g.* Cl^−^ regulates the osmotic pressure in the cell and Ca^2+^ is essential for intracellular signaling.^1^ They have also possibly been vital for the emergence of life on Earth: the prebiotic condensation reaction where the amino acids combine into peptides is not thermodynamically favorable in the presence of water.^2^ Deliquescent salts like CaCl_2_ are suggested to mediate the oligomerization *via* wet-dry cycling.^3,4^ Furthermore, these inorganic ions participate in various atmospheric processes from ozone depletion^5^ to cloud condensation.^6^ Ca^2+^ and Cl^−^ form a large portion of water-soluble ions present in the atmospheric aerosols, with seasonal and geographical variation.^7–9^ In these biological, prebiotic, and atmospheric processes, the presence and interaction with water is central. CaCl_2_ is an exceptional inorganic compound that grows hygroscopically from CaCl_2_·2H_2_O to CaCl_2_·6H_2_O until 20% RH, rather than experiencing deliquescence.^10–12^ CaCl_2_ deliquescence starts from 20% RH after the hydrate phase change.^13^ According to a recent study by Gough *et al.*,^14^ upon drying, CaCl_2_ can stay as a metastable supersaturated brine at RH values well below 10%. The same study reported also that the low temperature (223–273 K) deliquescence point of CaCl_2_ particles in the micrometer size regime depends strongly on the crystal structure of the particle, being on average 15.8 ± 3.5% RH for dihydrate and 63.3 ± 12.5% RH for hexahydrate forms. The efflorescence and deliquescence points of salts have been reported to be size-dependent, for example NaCl particles with dry diameters smaller than 40 nm deviate from macroscopic behavior by exhibiting increasing deliquescence relative humidity (DRH) and efflorescence relative humidity (ERH) with decreasing particle size.^15^ To the best of our knowledge, similar size-dependent data does not exist for CaCl_2_ particles, but in contrast to pure NaCl, the data reported for marine chloride mixtures (including Ca^2+^ as one of the cations) reports lower DRH and ERH values for 100 nm particles compared to supermicrometer particles.^16^ The reason for such behavior remained unclear. Especially while studying particles generated from aerosolized suspensions or solutions, drying of the aerosol stream is an important (pre)conditioning step. It is often performed using diffusion dryers, and the state of the aerosol particles are concluded from a RH measurement of the particle stream. However, water may remain in the particles even if they are dried, which would lead to inaccuracies in their dry-size determination and subsequent conditioning.^17^ Thus, from this point of view as well it is important to probe the state and chemistry of nanoparticles after drying the aerosol with diffusion dryers to different RHs.

In this work, we utilize X-ray absorption spectroscopy (XAS) to study the hydration state of atomizer generated free-flying CaCl_2_-NPs in the 100 nm size regime. XAS is a widely utilized element specific probe of the electronic structure and local chemical environment.^18–21^ The development of tuneable and high brilliance light sources like synchrotrons and free-electron lasers opens new avenues for the study of dilute systems using XAS. It has been applied to a variety of non-supported microparticles and NPs, such as electrodynamically trapped SiO_2_,^22^ Au-coated SiO_2_,^23^ levitating droplets,^24^*in situ* generated soot,^25^ and atomized salt solutions.^26,27^ Compared to X-ray-based photoelectron and photoabsorption spectroscopies nowadays available also with RH control for deposited nanoparticles^28^ and salt surfaces,^29^ using a continuously renewed nanoparticle beam we ensure that there are no substrate effects, radiation induced changes, and no further growth or change of particles after deposition. Here we apply XAS to study the hydration state of CaCl_2_-NPs by comparing XAS spectra of NPs generated at different RH to those obtained from water in different states, aqueous CaCl_2_ solution, and solid CaCl_2_. Ca 2p and Cl 2p XAS spectra were found to be insensitive to changing RH, albeit O 1s indicated that liquid-like water was present in particles. Moreover, we added phenylalanine (Phe) to the solution as a model biomolecule, representing an amino acid with a hydrophobic side essential for many processes in the human body,^30,31^ and also found in atmosphere.^32–34^ By using X-ray photoelectron spectroscopy, we recently observed that the addition of an organic acid to an aerosolized salt solution can drastically change the surface composition of dried NPs.^35^ Here, we observed that Phe and CaCl_2_ create mixed hydrated NPs. The XAS recorded at C 1s and O 1s edges agree in shape with previously reported solid state spectra of Phe, but relative energy shifts in bound and continuum resonance energies indicate interaction with a high ion concentration solvent and shortening of bond lengths.

## 2 Experimental details

**Fig. 1:**
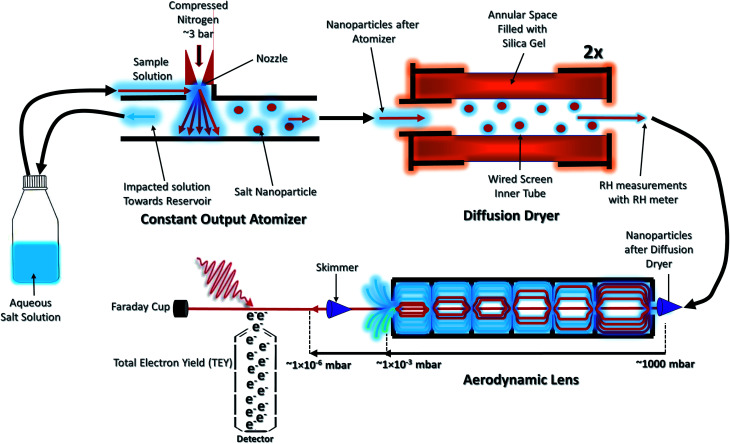
Schematic diagram to illustrate the generation of a free standing nanoparticle beam.

The NP experiments were carried out at the PLEIADES (Polarized Light source for Electron and Ion Analysis from Diluted Excited Species) beamline, SOLEIL synchrotron radiation facility.^36^ Detailed descriptions of the beamline instrumentation can be found in ref. 37–41. A schematic of the used experimental setup can be seen in Fig. 1. The time-of-flight (TOF) spectrometer of the beamline's permanent EPICEA end-station was used in Total Electron Yield (TEY) mode to measure the absorption spectra of the free-flying NPs.^36^ Photon energy calibration was done with carbon dioxide gas, using the 
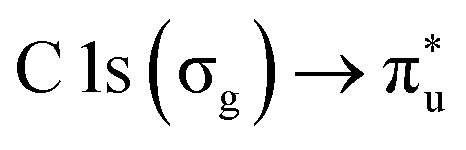
 resonance at 290.77 ± 0.05 eV (ref. 42 and 43) and the 
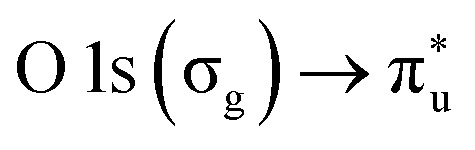
 resonance at 535.4 ± 0.1 eV.^43,44^ The photon energy offset is assumed to behave linearly between these two points. The XAS spectra are normalized to the photon flux (diode current) and particle flux (Faraday cup current). To ensure that the RH is maintained at the same range throughout the acquisition, the number of scans recorded for each spectrum was kept low (1–3).

The NPs were generated with a constant-output atomizer (no. 3076, TSI Inc, MS, US) using nitrogen as a carrier gas from 23 ± 0.1 mM aqueous solutions of CaCl_2_. A further 6 ± 0.1 mM of Phe was added to generate the mixed NPs. The solutions were prepared with CaCl_2_·6H_2_O purchased from Sigma Aldrich (Merck Group, St. Louis, US) and Phe purchased from Alfa Aesar (Thermo Fisher Scientific, Ward Hill, US), both with 99% purity. The humidity level was controlled with silica-filled diffusion dryers (model 3062, TSI Inc, MS, US). With two dryers a RH of 20% (measured with Sensirion AG, kit EK-H5) and with one drier a RH of about 50% was achieved. The highest RH measurement was obtained without drying. The RH measurements are taken after the diffusion dryers and before entering into the aerodynamic lens. The NPs were introduced inside the interaction chamber through an aerodynamic lens system,^36,45^ producing a narrow beam of NPs which intersected the synchrotron radiation beam at a right angle. A commercial differential mobility analyzer (DMA) together with a condensation particle counter (DMA 3081 & CPC 3786, TSI Inc, MS, US) was used to measure the particle size distributions.

For comparison of the NP spectra, XAS spectra of an aqueous CaCl_2_ solution at the Ca 2p and Cl 2p absorption edges were measured at the newly commissioned FlexPES (Flexible Photoelectron Spectroscopy) beamline at the MAX IV synchrotron radiation facility, Sweden. The XAS spectra were measured as partial electron yield (PEY), by collecting low kinetic energy electrons using a hemispherical electron analyzer (VG-Scienta R4000). The photon energies of the liquid jet experiment have not been calibrated to any standard reference, and the intensities have not been normalized to photon flux.

XAS spectra of solid CaCl_2_ (at Ca 2p,^46^ Cl 2p^47^) and H_2_O (ice, liquid, and gas at O 1s^48,49^) are taken from literature and were re-plotted with the help of online software.^50^ The XAS spectra of solid Phe (at C 1s, and O 1s) are taken from Zubavichus *et al.*,^51^ where the data files are given as supplementary material.

## 3 Results & discussion

### 3.1 Particle size analysis

**Fig. 2:**
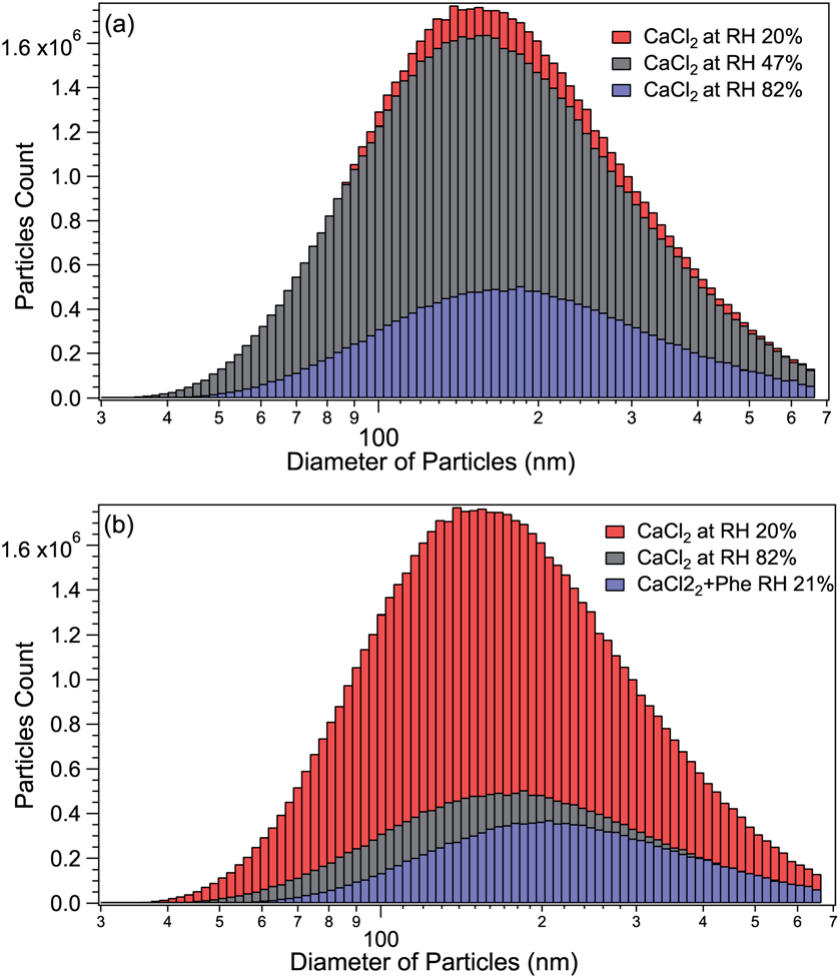
(a) Comparison of CaCl_2_-NPs average particle sizes at different humidity levels. (b) Comparison of CaCl_2_-NPs with and without Phe (the particle count rate is presented in particles per cm^3^ (d*N*/dlog *D*)).

The size distributions of CaCl_2_-NPs at different RH and of mixed CaCl_2_ + Phe-NPs measured with the DMA are presented in Fig. 2. Table 1 summarises the results. The aerodynamic diameter of CaCl_2_-NPs increases as a function of RH and upon the addition of Phe. The significant increase in particle size (150 nm to 180 nm) indicates a corresponding decrease in the internal concentration of the CaCl_2_-NPs. The number density of NPs decreases with increasing relative humidity. This decrease can indicate that the more humid stream contains larger droplets or their agglomerates, which are lost to the impactor or in the DMA cavity.^52,53^ The particle number density was lowered also with the addition of Phe.

**Table 1:** Comparison of the average particle size of CaCl_2_-NPs at different % RH levels and with added Phe

Salt	Number of dryers	RH%	Average particle size (nm)
CaCl_2_	0	82	180
CaCl_2_	1	47	165
CaCl_2_	2	20	150
CaCl_2_ + Phe	2	21	200

### 3.2 O 1s XAS – estimation of presence and state of water

**Fig. 3:**
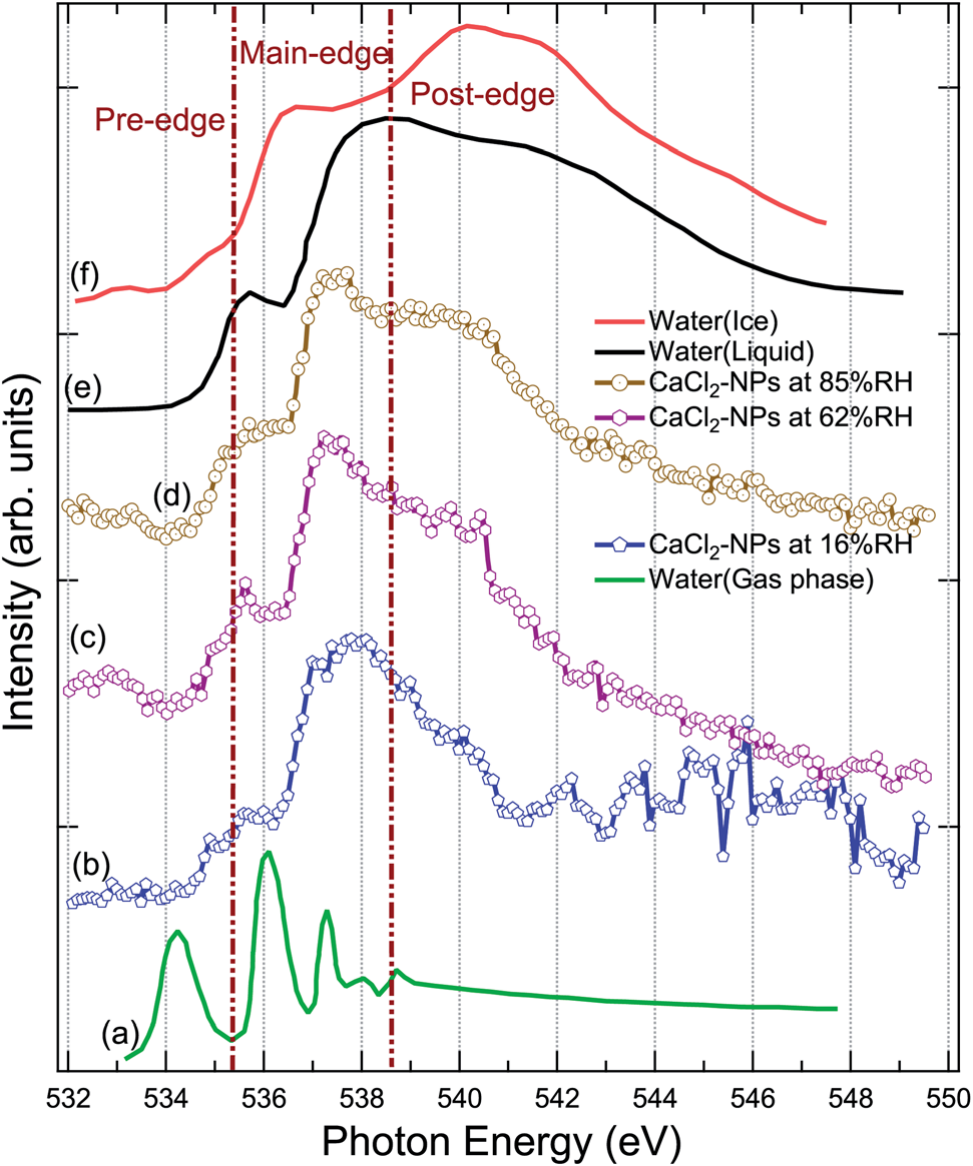
Comparison of the X-ray absorption spectrum of pure water in (a) gas, (e) liquid, (f) solid (ice) state at O 1s replotted from Fransson *et al.*,^48^ (b) CaCl_2_-NPs at 16% RH, (c) CaCl_2_-NPs at 62% RH, and (d) CaCl_2_-NPs 85% RH at O 1s.

XAS spectra were measured at the O 1s edge to characterise the presence and state of water in the particles. The NP spectra obtained at 16%, 62%, and 85% RH are shown in Fig. 3, together with XAS spectra of ice, liquid and gas-phase water replotted from Fransson *et al.*^48^ The pre-edge feature in the liquid water spectrum has been interpreted to indicate distortion in the hydrogen bonding network between water molecules,^48,49,54^ whereas the strong post-edge feature in ice originates from long-range tetrahedral structure.^18,55,56^ The NP spectra do not fully resemble the spectrum of solid nor liquid water, but are evidently more liquid like in all three regions (pre-edge, main-edge & post-edge), implying the absence of long-range tetrahedral networks. The main edge feature especially at 62 and 85% RHs show a sharper feature than liquid water. The increase in main edge at the cost of decrease in post-edge intensity has been previously reported to be related to the increased single-donor water configurations induced by the ions in the aqueous solution,^57^ and thus, the discrepancies to pure liquid water, namely at ∼538 eV and at ∼540 eV, are likely related to ions distorting the network of water molecules.^58,59^ From high to low % RH, the increase in the intensities of pre-edge and main-edge along with a decrease in the post-edge imply that the water content is reduced in the particles.^58^ A more detailed description of the structure and amount of water in the particles could be obtained by comparing their spectra to O 1s XAS spectrum of CaCl_2_·6(H_2_O), but unfortunately, we were not able to find that from the literature. Two scans were recorded for each NP spectra. The 16% RH NP spectrum suffers from instability of the particle beam due to the start of the clogging of the flow limiting orifice at the end of the second scan, which is seen as large fluctuations of signal at high energies of the post-edge region.

The % RH was measured from the aerosol stream before it enters the aerodynamic lens through a limiting orifice. Evaporation of water takes place when the NPs travel in vacuum to the interaction region. For a similar aerodynamic lens setup, Chang *et al.*^60^ estimated that evaporative cooling reduces the diameter of 100 nm water droplets to roughly 94 nm and the temperature of the water droplets is about 193 K, leaving them in a supercooled state. This estimation supports also our observation that CaCl_2_-NPs can have water in liquid state. However, based on O 1s XAS, Kostko *et al.*^27^ concluded that water is in both liquid and ice form in NPs produced *via* aerosolization of 38 mM NaI solution. They measured XAS using a Velocity Map Imaging (VMI) spectrometer collecting the partial electron signal from electrons with kinetic energy below 10 eV. Based on models for electron inelastic mean free path (IMFP) in water, the probing depth when collecting <10 eV electrons is more than 10 nm.^61^ In our experiment also higher kinetic energy electrons are collected, including the resonant and normal Auger electrons (kinetic energies > 500 eV). The IMFP for 500 eV electrons in water is 2–5 nm,^61–63^ and therefore our experiment can be somewhat more surface sensitive compared to the measurement by Kostko *et al.* using a VMI spectrometer.

To conclude, the results indicate the presence of liquid or liquid-like water within the probe depth in the CaCl_2_-NPs, the amount of which increases with higher % RH values.

### 3.3 Ca 2p XAS – decrease in crystal field splitting with water

**Fig. 4:**
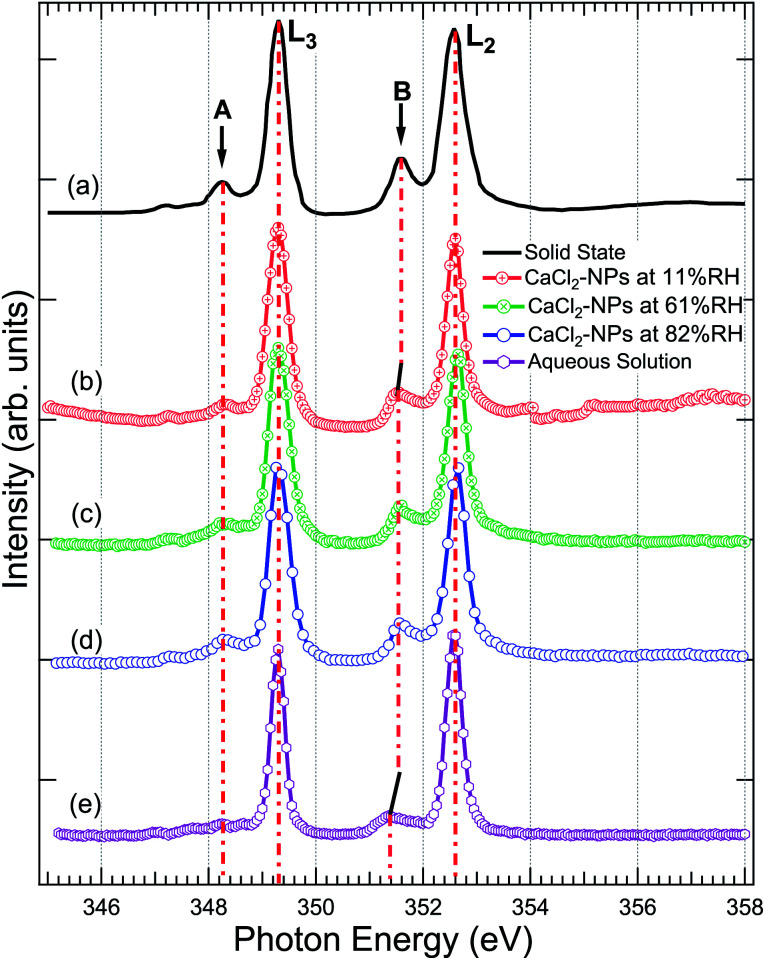
The X-ray absorption spectrum at Ca 2p of (a) powdered CaCl_2_·2H_2_O (replotted from Naftel *et al.*^46^) (b) CaCl_2_-NPs at 11% RH, (c) CaCl_2_-NPs at 61% RH, (d) CaCl_2_-NPs at 82% RH, and (e) CaCl_2_-aqueous solution. The solid and aqueous state spectra have been shifted to match the main 2p_3/2_ → 3d peak at 349.1 eV in NPs for easier comparison.

Fig. 4 shows a comparison of the Ca 2p X-ray absorption spectra of CaCl_2_, obtained from solid,^46^ aqueous solution, and non-supported NPs at different % RH levels. The overall shape of all the spectra is similar, exhibiting transitions from the 2p_1/2_(L_2_) and 2p_3/2_(L_3_) orbitals to the 3d orbital^64^ which are further split by crystal field effects. The two main peaks are assigned as 3d electrons in t_2g_ and the two smaller peaks (labeled “A” and “B”) in e_g_ symmetry in the first coordination sphere.^65,66^ According to calculations carried out by De Groot *et al.*^67^ the crystal field effects arise from octahedral symmetry (coordination number = 6. Although, in liquid water Ca is coordinated to 6–8 water molecules^58^) with a positive crystal field parameter. The features A and B are less pronounced in the aqueous state compared to the spectrum obtained from the solid.^68^ This indicates a decrease in the crystal field due to coordinated water molecules with specific geometric arrangements being dominated by steric effects.^69^ The crystal field effects in the CaCl_2_-NPs seem to be in between the solid and aqueous solution, indicating decreasing Ca^2+^–Cl^−^ ion interaction or increasing distorted solvation shells^70^ in NPs compared to solid state and aqueous solution. In addition to a decrease in relative intensity between A and L_3_, and B and L_2_, there is a small shift of the crystal field split peak B towards a lower photon energy in relation to the L_2_ peak from solid to NPs, and from NPs to aqueous solution XAS spectra, as shown in Fig. 4. The Ca L-edge spectrum of CaCl_2_·6H_2_O was not found in the literature, but the K-edge XAS spectra of the CaCl_2_ aqueous solution and CaCl_2_·6H_2_O are similar apart from a more intense pre-edge for CaCl_2_·6H_2_O.^71^ They differ from CaCl_2_ and CaCl_2_·2H_2_O, which in turn are similar to each other.^71^ The calculations done for aqueous solutions of CaCl_2_ by Badyal *et al.*^72^ showed that high concentrations up to 6 M had minimal effects on the coordination geometry of the first solvation shell of Ca^2+^, and there was no Ca^2+^–Cl^−^ contact ion interaction. The concentration did not show any effect on the solvent coordination of Cl^−^ either. Calculations of Megyes *et al.*^70^ found that at 4 to 6 M concentrations the solvation shell gets more distorted, but they neither found signs of ion interaction. It seems that in our case, even upon drying the NPs by two dryers and by further evaporation of water in-flight in vacuum, the water content at the surface is sufficient to avoid Ca^2+^–Cl^−^ contact ion interaction, and the first solvation shell around Ca^2+^ ions remains unchanged despite the % RH in which the NPs were prepared.

### 3.4 Cl 2p XAS – indication of solvated Cl^−^ ions

**Fig. 5:**
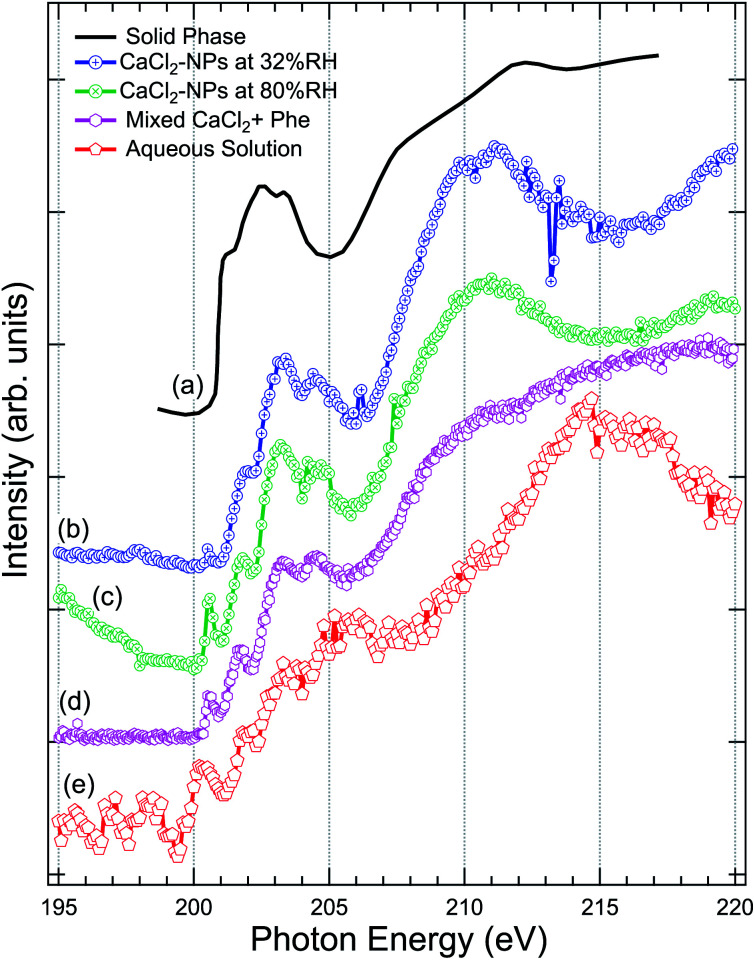
Comparison of the X-ray absorption spectra at Cl 2p, of (a) pure CaCl_2_ (thin film solid phase) from Sato *et al.*,^47^ (b) CaCl_2_-NPs at 32% RH, (c) CaCl_2_-NPs at 80% RH, (d) mixed CaCl_2_ + Phe, and (e) CaCl_2_-aqueous solution.

The Cl 2p XAS spectra of CaCl_2_-NPs measured at different % RH are shown in Fig. 5 together with spectra of aqueous solution and *in situ* evaporated, dried solid film CaCl_2_ (from Sato *et al.*^47^).

Concerning the solid CaCl_2_, based on the temperature independence and shape of the spectrum with less distinctive peaks after a sharp onset around 201 eV, Sato *et al.* concluded that their sample was amorphous.^47^ The aqueous spectrum suffers from strong noise, but we can get an overall idea of the shape of the spectrum: a pre-peak slightly above 200 eV, main absorption edge at around 202 eV followed by strong enhancement of the signal starting from around 211 eV. The worse signal-to-noise ratio in low kinetic energy PEY liquid jet Cl 2p XAS compared to Ca 2p XAS can be at least partially explained by the fact that the kinetic energies of Auger electrons are much lower in case of Cl 2p compared to Ca 2p, and thus the amount of subsequent secondary low kinetic energy electrons per ionization is correspondingly lower. The NP spectra agree in general shape with the solid state spectrum, but features between 200 and 205 eV seem to be better resolved in NPs. In addition, there is a sharp pre-peak around 200.5 eV in 80% RH (with some ambiguity also in the 32% RH spectrum) and the aqueous solution spectra, which is missing from the amorphous solid spectrum. The pre-peak structure resembles the sharp peak observed for Cl^−^ ions in an experiment by Bournel *et al.*^73^ and Parent *et al.*^74^ where HCl was dissociatively adsorbed at 120 K on a crystalline water-ice film. Thus, as this peak is present in the high % RH spectra for the NPs and the aqueous solution and not in the amorphous solid spectrum, it is an indication of change in local coordination geometry and formation of dissolved Cl^−^ species.

The aqueous spectrum shows very similar post-edge behaviour as the Cl^−^ ion TEY spectrum,^74^ with a maximum at 215 eV. In CaCl_2_-NP spectra this maximum is shifted towards lower photon energies, reaching maximum at 211 eV and minimum at 215 eV. Amorphous solid does not exhibit a clear maximum but rather a broad continuum starting from 205 eV. The post-edge region is dominated by complex multi-electron and multiple scattering processes, and theoretical modelling would be needed to analyse these changes further. In general, the behaviour of CaCl_2_-NP Cl 2p edge spectra is in line with the observations at O 1s and Ca 2p edges, supporting the picture that the probed surface layer of CaCl_2_-NPs consists of Ca^2+^ and Cl^−^ ions separated by solvent water molecules.

### 3.5 Mixed hydrated NPs of CaCl_2_ and Phe

The production of mixed CaCl_2_ + Phe NPs from aerosolized solution of CaCl_2_ and Phe was probed using Cl 2p, C 1s, and O 1s XAS. Cl 2p XAS spectrum of CaCl_2_ + Phe NPs measured with RH < 25% is presented in Fig. 5. This spectrum also shows a pre-peak like aqueous CaCl_2_ and humid CaCl_2_-NPs. CaCl_2_ + Phe NPs show similar broad post-edge structure as the amorphous solid spectrum, but differs from CaCl_2_-NP spectra. The change in the post-edge back-scattering region upon addition of Phe can indicate that some Cl^−^ anions are in the vicinity of Phe, which has a positively charged amine group in the zwitterionic form.

**Fig. 6:**
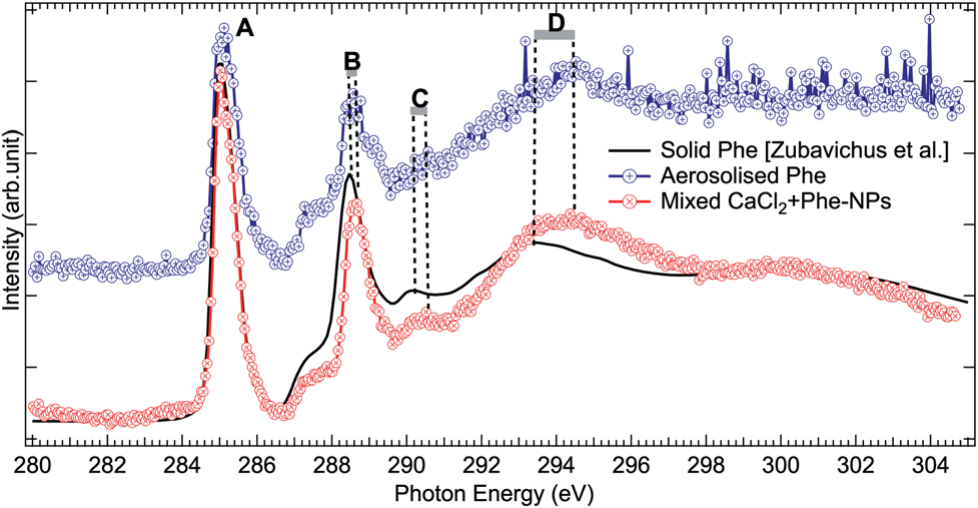
Comparison of C 1s XAS from thin solid film Phe (from Zubavichus *et al.*,^51^ black solid line), from aerosolized pure Phe aqueous solution (blue solid line and circles), and from mixed CaCl_2_ + Phe NPs (red line with circles). The spectra have been normalized to the highest peak by shifting the solid state spectrum. Letters (A)–(D) indicate the main features, whose shifts are discussed in the text.

The C 1s XAS of CaCl_2_ + Phe NPs and aerosolized aqueous solution of pure Phe are compared to a solvent-free polycrystalline powder film of Phe^51^ in Fig. 6. Three consecutive scans were recorded for nanoparticle and pure Phe spectra. The signal of aerosolized pure Phe solution was remarkably lower than from aerosolized mixed CaCl_2_ and Phe solution, indicating that Phe most likely does not form large agglomerates which would be effectively focused with the aerodynamic lens (highest transmission for approximately 100 nm diameter particles), and in fact the signal of pure Phe represents mostly isolated molecules or small clusters of Phe. Thus, while aerosolization of mixed CaCl_2_ and Phe solution may produce also pure Phe aerosols, their contribution to the CaCl_2_ + Phe NPs spectra is very small. Despite the small concentration of Phe in the initial solution (6 mM), the signal at C 1s level in CaCl_2_ + Phe NPs is relatively strong. Even if we cannot deduce any absolute values of absorbance, Phe seemed to be well abundant within the probing depth. Based on observations of pure CaCl_2_ NPs, the surface layer consists of liquid-like water, and since Phe has a hydrophobic benzene ring, it may lead to the enrichment of the molecule on surface with benzene rings avoiding the water.

All the C 1s spectra have a sharp, slightly asymmetric feature at 285.02 eV (solid at 285.13 eV), labelled “A” in Fig. 6, originating from the C 1s → π* transition in the benzene ring. The second sharp peak, labelled “B” in Fig. 6, is located at 288.57 eV for aerosolized Phe, at 288.67 eV for mixed NPs, and at 288.58 eV for solid Phe (note that spectra in Fig. 6 have been aligned with respect to peak “A”). This peak has been assigned to the C 1s → π* transition in C=O.^51,75–77^ It is noteworthy that the energy separation between peaks A and B is the same for the solid sample and aerosolized Phe, but larger in the CaCl_2_ + Phe NPs case. The difference in energy separation with respect to peak A becomes even larger when looking at the features “C” and “D”: while the statistics of the aerosolized Phe makes it difficult to define these features accurately, there is no doubt that a clear shift between the solid and CaCl_2_ + Phe NPs exists. The feature C at 290.47 eV for CaCl_2_ + Phe NPs and at 290.28 eV in solid Phe has been assigned to mostly originate from transitions from α-carbon C 1s to σ*-type of orbitals, oriented between the C and N atoms. The broad feature D with a maximum at around 294.47 eV for mixed NPs and at 293.48 eV for solid Phe has been assigned to C 1s → σ* transitions in benzene rings.^78^ In gas phase Phe, this resonance has been found at same energy as in solid state.^78^ Changes between XAS of solid Phe and CaCl_2_ + Phe NPs in the energy range of bound state transitions indicate interaction with solvent with high ion concentration, whereas blue shift in continuum σ* resonances of benzene have been shown to qualitatively correlate with shortening in bond length.^79^

**Fig. 7:**
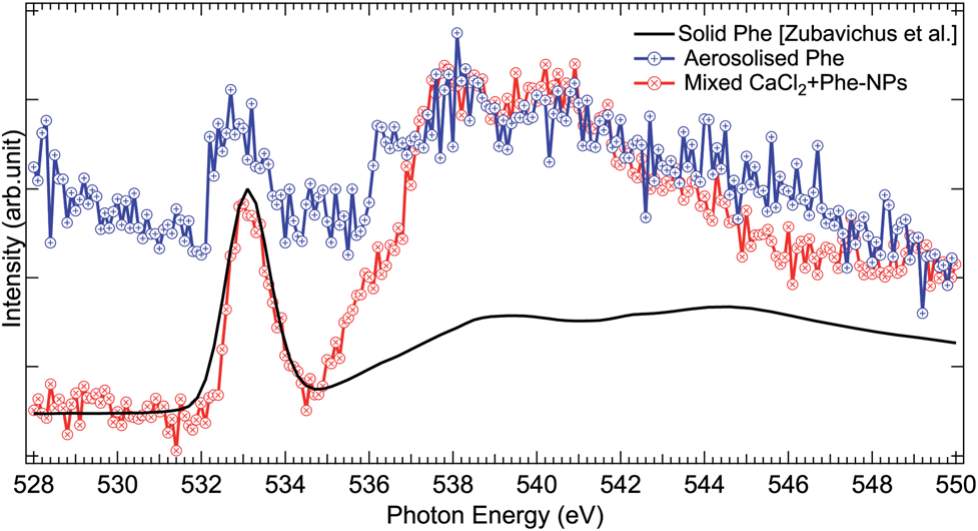
Comparison of O 1s XAS spectra from thin solid film Phe (from Zubavichus *et al.*,^51^ black solid line), aerosolized pure Phe aqueous solution (red solid line and circles), and mixed CaCl_2_ + Phe-NPs (black line with circles). The spectra have been normalized to the highest peak by shifting the solid state spectrum.

The O 1s absorption spectra (three scans each) of pure Phe and CaCl_2_ + Phe NPs were also measured (as shown in Fig. 7). The first prominent feature at 533.1 eV correspond to the O 1s → π* antibonding transition in the C=O group. Several overlapping O 1s → σ* transitions contribute in the region 535–550 eV of Phe.^77^ CaCl_2_ + Phe NPs seem to have significant contribution from the liquid water-like spectrum, and the region above 535 eV is a superposition of water and Phe spectrum. The aerosolized Phe spectrum is very weak, and can have a contribution from water as well.

To conclude, the aerosolization of mixed CaCl_2_ and Phe solution can produce NPs with liquid-like water within the probing depth and strong X-ray absorption signal from Phe. The C 1s XAS spectrum from CaCl_2_ + Phe NPs is different from solid Phe, originating from solvent effects and high salt concentration in the NPs.

## 4 Conclusions

Free-flying CaCl_2_-NPs were produced from an aqueous CaCl_2_ solution using an atomizer and the effect of RH of the aerosol stream (∼11–85%) on the CaCl_2_-NPs was investigated. This study demonstrates novel use of soft X-ray absorption spectroscopy to investigate hydration of free-flying salt NPs and their mixtures with amino acids.

The O 1s XAS spectra show that within the 2–5 nm probing depth, water is present in a distorted liquid-like state throughout the investigated RH range. The increase in the amount of free water as a function of RH would change the relative intensity of pre-, main-, and post-edge regions, and we observe a slight relative increase in post-edge region intensity when RH gets higher. The Ca^2+^ and Cl^−^ ions remain fully hydrated and no crystalline salt is observed in the NPs, and no major changes are observed in the Ca 2p & Cl 2p edges implying similar primary hydration layers at all RH-values.

Soft X-ray absorption spectroscopy was also carried out for mixed nanoparticles aerosolized from a solution of CaCl_2_ and hydrophobic amino acid Phe. The differences in post-edge features in the Cl 2p edge of CaCl_2_ + Phe NPs compared to CaCl_2_ NPs can originate from the presence of Cl^−^ in vicinity of Phe. The energy shifts of the spectral features at the C 1s absorption edge were observed for both aerosolized Phe and mixed CaCl_2_ + Phe compared to solid Phe. The blue shift of the continuum resonance region indicates changes in the bond lengths of Phe from solid to aerosolized Phe and mixed CaCl_2_ + Phe. Further work is needed to understand how Phe is oriented on the surface of the hydrated CaCl_2_-NPs, but our present work demonstrates that we can produce hydrated mixed aerosol particles as an interesting platform for such studies. In addition, future experiments with fully dried NPs and complementary techniques with larger probing depth such as high-kinetic energy X-ray photoelectron spectroscopy will provide further information on the overall structure of the CaCl_2_-NPs.

## Conflicts of interest

There are no conflicts to declare.

## Supplementary Material
